# Nitric Oxide-Dependent Pathways as Critical Factors in the Consequences and Recovery after Brain Ischemic Hypoxia

**DOI:** 10.3390/biom11081097

**Published:** 2021-07-26

**Authors:** Joanna M Wierońska, Paulina Cieślik, Leszek Kalinowski

**Affiliations:** 1 Maj Institute of Pharmacology, Polish Academy of Sciences, Smętna Street 12, 31-343 Kraków, Poland; wierons@if-pan.krakow.pl (J.M.W.); cieslik@if-pan.krakow.pl (P.C.); 2 Department of Medical Laboratory Diagnostics—Biobank Fahrenheit BBMRI.pl, Medical University of Gdansk, Debinki Street 7, 80-211 Gdansk, Poland; 3 Biobanking and Biomolecular Resources Research Infrastructure Poland (BBMRI.PL), Debinki Street 7, 80-211 Gdansk, Poland; 4 BioTechMed Center/Department of Mechanics of Materials and Structures, Gdansk University of Technology, Narutowicza 11/12, 80-223 Gdansk, Poland

**Keywords:** nitric oxide, cerebral ischemia, excitotoxicity, eNOS, nNOS, iNOS, oxidative/nitrosative stress, HIF-1α

## Abstract

Brain ischemia is one of the leading causes of disability and mortality worldwide. Nitric oxide (NO^•^), a molecule that is involved in the regulation of proper blood flow, vasodilation, neuronal and glial activity constitutes the crucial factor that contributes to the development of pathological changes after stroke. One of the early consequences of a sudden interruption in the cerebral blood flow is the massive production of reactive oxygen and nitrogen species (ROS/RNS) in neurons due to NO^•^ synthase uncoupling, which leads to neurotoxicity. Progression of apoptotic or necrotic neuronal damage activates reactive astrocytes and attracts microglia or lymphocytes to migrate to place of inflammation. Those inflammatory cells start to produce large amounts of inflammatory proteins, including pathological, inducible form of NOS (iNOS), which generates nitrosative stress that further contributes to brain tissue damage, forming vicious circle of detrimental processes in the late stage of ischemia. S-nitrosylation, hypoxia-inducible factor 1α (HIF-1α) and HIF-1α-dependent genes activated in reactive astrocytes play essential roles in this process. The review summarizes the roles of NO^•^-dependent pathways in the early and late aftermath of stroke and treatments based on the stimulation or inhibition of particular NO^•^ synthases and the stabilization of HIF-1α activity.

## 1. Brain Ischemic Stroke and the Role of NO Its Pathology

Insufficient blood flow to a tissue results in hypoxia (the lack of an adequate oxygen supply at the tissue level) or anoxia (the absence of oxygen). Acute arterial thrombus formation, chronic narrowing of a supply artery and arterial vasospasm are the most critical factors contributing to the local or generalized deprivation of oxygen, which results in ischemia. In the 50-year-old and older age group, brain stroke is the second leading cause of disability [1] and, after coronary artery disease, constitutes the second most common cause of death worldwide [2].

As shown in vivo in transient forebrain ischemia model in rats, the mechanisms of neuronal death following ischemia include both apoptosis, i.e., a form of programmed cell death, highly regulated and controlled process that involves events such as cell shrinkage, nuclear fragmentation, chromatin condensation, chromosomal DNA fragmentation [3] and necrosis, a form of traumatic cell death that results from the disruption of membrane integrity, which attracts leukocytes to accumulate around the necrotic cells and to release cytokines [3], inducing collateral damage thus enabling the healing of the tissue.

Among the variety of factors that contribute to the cellular events leading to ischemic neuronal death, the fundamental factors are N-methyl-D-aspartate receptor (NMDA)-induced excitotoxicity and NO^•^-dependent pathways which are functionally linked [4,5,6].

NO is formed by the oxidation of nitrogen and is biosynthesized endogenously from L-arginine, which is converted first to N-hydroxyl-arginine, then to L-citrulline and finally to NO in the presence of NADPH and tetrahydrobiopterin (BH_4_) as cofactors [7]. The main enzymatic target of NO^•^ is activation of guanylyl cyclase (GC) [8,9], which leads to cGMP production and the subsequent activation of a variety of proteins essential for a number of critical processes in the brain [9] amplifying the excitatory cell responses modulated by NMDA-dependent signaling [10].

At least three NOS isoforms are responsible for NO^•^ synthesis, including neuronal NO synthase (nNOS), which mediates the production of NO^•^ in neurons; the endothelial (eNOS) isoform, which is found on the inner surface of blood vessels. The activity of eNOS and nNOS, which are called constitutive NOS isoforms (cNOS), is dependent on Ca^2+^ and calmodulin complex, and the production of NO^•^ is initiated in response to elevations in intracellular Ca^2+^ levels triggered by both mechanical forces and substances circulating in the blood (e.g., glutamate, acetylcholine, ATP) [11,12].

In contrast to cNOS, the activity of inducible NO^•^ synthase (iNOS) is Ca^2+^-independent [12,13]. The enzyme is activated mostly in glial cells or leukocytes by certain cytokines, such as interferon-γ, tumor necrosis factor-α or interleukin-1β, in response to inflammation [12,13].

In the physiological state, glutamate activates the synaptic pool of NMDA receptors and is rapidly taken up from the synaptic space by presynaptic mechanisms and astrocytes. Opening of NMDA ion channels and subsequent Ca^2+^ influx into the neurons activates constitutive nitric oxide synthases (cNOS) to produce nitric oxide (NO) [4,5,6].

Under pathological conditions, due to the “uncoupling” of NOS activity from electron donation by NADPH and/or reduced availability of L-arginine or BH_4_, electrons are transferred from NADPH to flavins via the reductase domain, forming superoxide (O_2_^•^^−^), and other reactive oxygen species (ROS) [11,14]. NO^•^ reacts with O_2_^•^^−^ to form reactive nitrogen species (RNS) including the highly toxic peroxynitrite (ONOO^−^), leading to the production of other secondary components of nitroxidative stress, such as NO^2+^, NO_2_ and OH^•^, which initiate a cascade of redox reactions [11,14].

iNOS produces much greater amounts of NO^•^ than both eNOS and nNOS combined. Therefore, iNOS is often referred to as the “pathological” form of NO^•^ synthase, as it may promote the production of ONOO^−^ and subsequently highly reactive hydroxyl radicals [14,15].

**Figure 1:**
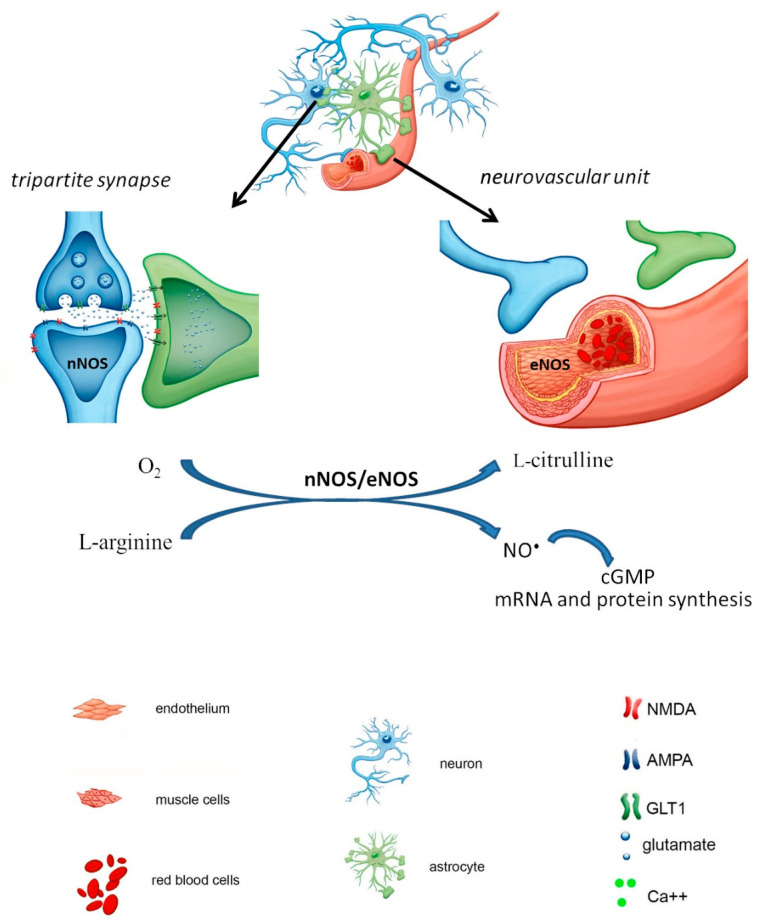
The activity of nNOS/eNOS dependent pathways in the brain under physiological conditions: glutamate released from the presynaptic neuron is rapidly uptaken by the astrocyte; nNOS and eNOS are kept at the normal level and both enzymes synthesize NO^•^ which activates cGMP synthesis.

The key role of NO^•^ in the brain is its impact on the components of the neurovascular unit. Thus, the vasodilation or vasoconstriction of blood vessels, neuronal excitability and glial cell functioning are dependent on NO^•^ signaling mediated by constitutive NOS isoforms (nNOS and eNOS) (Figure 1) [11,16].

The distribution and concentration of NO^•^ in brain tissue change after ischemic episode and differs both temporally and spatially postinjury. Time-dependent changes induced by ischemia are related in particular to the activity of individual NOS isoforms, and in general, NO^•^ produced by nNOS or iNOS plays detrimental role [14]; while eNOS is neuroprotective [17]. The expression of nNOS increases rapidly, in parallel with membrane depolarization and Ca^2+^ elevation in the cells, while massive production of iNOS is observed several hours after ischemic episode [14]. Such correlations were observed predominantly in the animal model of cerebral ischemia-reperfusion based on middle cerebral artery occlusion (MCAO), which is usually used and is believed to reproduce the pattern of ischemic brain damage observed in many humans ischemic stroke patients [18,19].

Based on these pre-clinical observations the early and late stage of the ischemia, alternatively called early and late neuronal post-ischemic damage, has been described in several research papers [14,20,21,22,23], and in reviews [24]. Clinical studies concerning the progression of ischemia damage and the rsole of NO^•^ can be found elsewhere [25,26,27,28,29].

### 1.1. Early Stage of the Ischemia (Early Neuronal Damage)

The obstruction of blood flow by the clot dramatically reduces glucose and oxygen supply in ischemic brain region and triggers ischemic cascades, which include accumulation of lactate and malfunction of ion pumps (Na^+^/K^+^-ATPase and Ca^2+^/H-ATPase), subsequently inducing membrane depolarization and calcium ion (Ca^2+^) overload. Membrane depolarization causes the release of excitotoxic amino acid glutamate and its translocation into the extracellular compartment. The reversal of the activity of glutamate transport proteins prevents glutamate reuptake which results in robust increase in glutamate level and activation of extrasynaptic NMDA receptors [4,16,30,31].

In vivo studies show, that in the MCAO model in rats, immediate early genes (c-fos, c-jun) are activated by protein kinases and other second messengers shortly after the onset of ischemia.

The synthesis of NO^•^ through nNOS is mainly related to calcium overload induced by glutamate in ischemic neurons [32]. The exacerbated activation of nNOS and the excess NO^•^ production due to uncoupled state, contributes to neurotoxicity and neurodegeneration by free radical formation [33,34,35].

**Figure 2:**
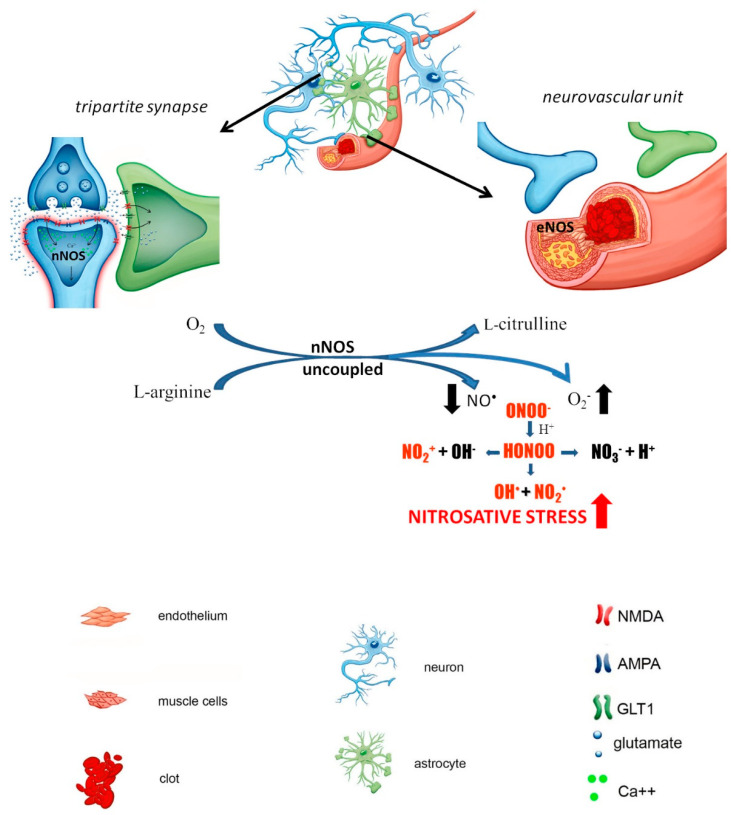
Early stage of the ischemia (early neuronal damage (0–6 h after ischemic episode): when the clot is formed due to atherosclerotic plaque, under restriction of oxygen supply, the glutamate released by presynaptic neuron accumulates in the synaptic cleft due to reversed activity of GLT1 transporter in the astrocyte; this leads to overactivation of postsynaptic neuron, overactivation of voltage-dependent calcium channels and Ca^2+^ influx into postsynaptic neuron, that leads to overactivation of nNOS and its uncoupling, and accumulation of ROS/RNS, instead of cGMP.

Neurons are particularly sensitive to stress caused by ROS/RNS overproduction because of the relatively low levels of antioxidants compared with other cells. Physiologically, basal ROS/RNS production in neurons is constitutively generated by mitochondrial metabolism, which is higher than that in other cells due to the necessity to maintain neuronal circuit activity and synaptic transmission. Excessive nNOS activation under hyperactivation of NMDA receptors results in massive and uncontrolled generation of NO^•^ followed by the downstream formation of ROS/RNS, resulting in neuroinflammation and nitro oxidative burst. The prolonged destabilization of nNOS contributes to neurotoxicity and the potentiation of ischemic damage (Figure 2) [36,37]. Genetically engineered mice that overexpress the free radical scavenger, superoxide dismutase, have less edema formation than wild-type littermates confirming the detrimental role of free radicals in ischemia pathology [38,39,40].

A second glutamate-independent rise in Ca^2+^ is observed 2–3 h after an ischemic episode, further contributing to the activation of Ca^2+^-dependent enzymes (except NOS also proteases, phospholipases, cyclooxygenases, endonucleases) and leading to irreversible changes that promote the apoptotic and necrotic death of neurons [41,42,43,44,45].

In animal studies with the use of nNOS^−/−^ knockout (KO) mice, in the model of cerebral ischemia-reperfusion, the smaller infarct size comparing to their wild-type littermates was observed. Relative cerebral blood flow after reperfusion was also higher and the levels of nitrates was significantly decreased in nNOS^−/−^ mice [46,47]. No 3-nitrotyrosine immunoreactivity, a marker for ONOO^−^ formation associated with cell death, was reported in mutant mice deficient in nNOS activity, which were subjected to reversible MCAO [16].

In contrast to nNOS, NO^•^ generated by eNOS expressed in the endothelial cells has been suggested to have beneficial effects [17]. Here, eNOS not only promotes vascular dilation but also increases vascular smooth muscle cell proliferation and migration, and thereby enhances arteriogenesis after stroke [48].

In this case, eNOS deficient mice suffered from more severe ischemia-reperfusion injury in MCAO model, significantly reduced cerebral blood flow which subsequently resulted in greater infarct size [46,47,49].

The results of in vivo studies clearly suggest that the generation of NO^•^ and its neurotoxicity after reperfusion is closely related with the activity of nNOS. Additionally, nNOS-derived NO^•^ may play a major role in early blood-brain barrier BBB disruption following transient focal cerebral ischemia [50].

### 1.2. Late Stage of the Ischemia (Delayed Neuronal Damage)

Immediate early gene products activate transcription sites on the cytokine genes such as tumor necrosis factor-α (TNF-α) or interleukin-1β (IL-1β) that appear within hours after stroke. Appearance of cytokines attracts inflammatory cells such as neutrophils, that appear 4 to 6 h after a stroke [51,52]. P-selectin, E-selectin, vascular cell adhesion molecule (VCAM) and intercellular adhesion molecule (ICAM) appear on the vessels with different time courses, but the ultimate effect is that neutrophils rolling along the vessel attach and cross into the brain. At later times macrophages become the predominate cell type in the injured site [15,53].

The role of glia cells (astrocytes and microglia) is pivotal in progressing cerebral ischemia. Astrocytes play critical role BBB integrity and the maintenance of extracellular ion homeostasis by buffering excitatory transmitters released by neurons and producing trophic factors that support neuronal growth and survival. In response to pathological situations in surrounding tissue such as ischemic injury astrocytes undergo morphological, molecular and functional changes and become reactive astrocytes [54] The changes associated with the reactive state can directly impact synaptic transmission and neuronal circuit activity, thereby potentially contributing to the pathological changes observed after ischemia [55].

On the other hand, resting microglia in the mature brain is a powerful weapon under pathological activation. Following ischemia, as a result of rapid neuronal death, microglia migrates to damaged tissue to exert a neuroprotective effect by clearing dead tissue, inhibiting cytotoxic neuronal damage and releasing neuroprotective growth factors. However, microglial activation triggers iNOS production, which, together with the parallel appearance of reactive astrocytes, results in massive iNOS expression observed relatively late after an ischemic episode (after approximately 24 h) and generation of ROS/RNS [51,55,56,57,58]. In some brain areas, astrocytes also express nNOS, which may be tonically active after chain reactions initiated by ischemia. However, some contrary reports indicate the minor role of microglial iNOS in mediating brain injury after stroke [59].

In contrast to nNOS, the induction of iNOS expression begins 12 h after induction of ischemia, increases progressively over time and reaches maximal levels after approximately 24–48 h as shown in transient MCAO model in animals [60]. The other in vivo studies confirm that NO derived from iNOS appears to contribute to neurotoxicity after ischemic stroke. The infarct and the motor deficits produced by MCAO were smaller in iNOS knockouts than in wild-type mice confirming that iNOS-derived NO^•^ is one of the factors contributing to the expansion of the brain damage that occurs in the late stage of post-ischemic period [61]. Furthermore, the reduction of infarct size and improvement of neurological deficits was not observed up to 24 h after MCAO, indicating that iNOS does not participate in the initiation of ischemic brain damage [61].

**Figure 3:**
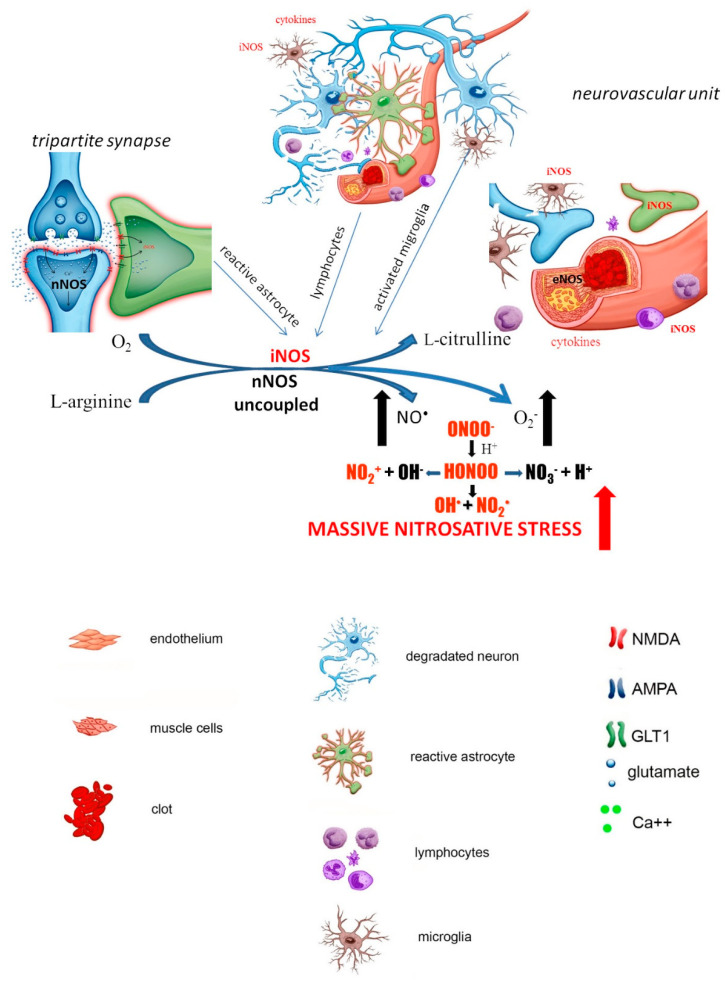
Late stage of the ischemia (delayed neuronal damage (begins several hours after episode and reaches maximal level after 24–48 h): parts of dead neurons attract leucocytes and microglia, which accumulate in the place of the stroke releasing cytokines that start to induce the production of iNOS in these cells. Additionally, astrocytes undergo morphological, molecular and functional remodeling in response to injury and also becoming a source of proinflammatory cytokines and iNOS. Thus, the cascade of events triggered by ischemia leads to massive production of cytokines and accumulation of proinflammatory cells with high activity of iNOS. Under these circumstances, in the strong deprivation of tetrahydrobiopterin (BH_4_) and/or substrate L-arginine, all uncoupled NOS isoforms generate simultaneously high amounts of both NO^•^ and O_2_^•^^−^ favoring production of ONOO^−^. The uncoupling of NOS isoforms can be additionally potentiated by O_2_^•^^−^ overproduction from other enzymatic sources in the cells accumulated in ischemic lesion, such as upregulated NAD(P)H-oxidase. It finally results in the generation of massive ROS/RNS and a vicious molecular circle is formed which aggravates the neurodegeneration process.

Pathological glial activation escapes endogenous control and turns into an autoaggressive pathomechanism that contributes to secondary neuronal damage occurring after brain ischemia [58]. Leukocytes accumulating at the site of neuronal necrosis act as an additional barrier to this cascade of destructive events by secreting cytokines, including interferon-γ and interleukins (Figure 3).

Overall increased activity of nNOS/iNOS isoforms results in the subsequent production of NO^•^ in neurons, glia, neutrophils and the rest inflammatory cells inducing the release of inflammatory factors that promote cytotoxicity, including the increased activation of cell adhesion molecules, cytokines, TNFα and matrix metalloproteinases (MMPs) [62]. TNFα, caspases and MMPs are particularly important in triggering apoptotic signals in cells and contribute to BBB damage, neurotoxic substance release, free radical generation, oxidative stress and brain edema [63,64]. As a result, after hours and days the infarct size expands due to excitatory amino acid release, loss of ion homeostasis, decreased pH, inflammation and edema, causing additional damage and apoptosis in the surrounding areas [65]. The strategy for post-ischemic neuroprotective therapies is to target the peri-infarct or penumbra region to prevent or rescue the spreading damage of the initial infarct.

Reassuming, ischemic brain injury involves a complex interaction between leukocytes, glia, neurons and the endothelium, which form a vicious chain of events that results in massive and continuous production of iNOS resulting is subsequent generation of ROS/RNS [59,60]. The pathological events associated with ischemic brain injury involve energy failure, oxidative stress, acidosis, disruption of ion homeostasis, neuronal cell excitotoxicity or inflammation and evolve progressively over time. The early and late stages of ischemia described above concern not only sequential NO^•^ production but also immunological responses (which are in part related to NO^•^ generation) described elsewhere in more details (for review see: [66,67]).

## 2. NO-Dependent Factors Aggravating Ischemic Cascade

In addition to guanylyl cyclase activation and subsequent initialization of cGMP production (or pathological generation of ROS/RNS), NO^•^-mediated processes control the functioning of many proteins and genes expression. Among them, s-nitrosylation and HIF-1α stabilization seem to be of importance in pathophysiology of brain ischemia.

### 2.1. S-Nitrosylation

The NO^•^-mediated S-nitrosylation process is a redox-based posttranslational modification that modulates protein function and activity. S-nitrosylation is the chemical reaction of an NO^•^ moiety with the sulfhydryl groups of target proteins, which leads to the formation of S-nitrosothiols (R-SNO), producing S-nitrosylated proteins (SNO-proteins) [68].

S-nitrosylation can occur both extracellularly and intracellularly [69]. The S-nitrosylation of the regulatory binding partners of transcription factors (TFs) (for example, HIF-1α) may impose an extranuclear influence on their activation, stability and nuclear targeting [69,70]. On the other hand, the S-nitrosylation of critical redox-sensitive Cys residues in the DNA-binding or allosteric sites of TFs invokes alterations in gene expression [71,72]. Finally, S-nitrosylation may regulate protein function by the covalent addition of an NO group to a cysteine thiol/sulfhydryl group (RSH or, more properly, thiolate anion, RS^−^) to form S-nitrosothiol derivatives (RS-NO), which is the most important aspect of the S-nitrosylation process [70]. The reaction is mediated by NO^•^-related species including NO^•^, NO^−^ (nitroxyl anion, which is NO^•^ with one additional electron) and NO^+^ (nitrosonium ion, which has one fewer electron than NO^•^) [70].

In addition to the process of S-nitrosylation, functional equivalents of NO^+^ can be transferred from one nitrosothiol to another in a process called transnitrosylation, whereby an NO^•^ moiety is transferred from a SNO-protein to a free thiol on another protein, which occurs when two proteins interact directly and possess appropriate redox potentials to allow electron transfer [73].

Aberrant S-nitrosylation occurs as a consequence of exacerbated nitrosative stress via the excessive production of NO^•^, which nitrosylates cysteine thiols with only partial SNO motifs or located more distant from the NO^•^ source [74]. These aberrantly S-nitrosylated proteins may contribute to pathological changes by triggering protein misfolding, mitochondrial dysfunction, transcriptional dysregulation, synaptic damage and neuronal injury [68]. In contrast, some SNO-proteins lose NO^•^ groups from their Cys thiols and undergo denitrosylation, which contributes to the regulation of the SNO signaling cascade [73]. Therefore, both S-nitrosylation and denitrosylation regulate protein activity and may be involved in pathological processes.

Under physiological conditions, nNOS is S-nitrosylated by NO^•^. The studies were performed both in vitro in HEK223 cell lines, cultured primary cortical neurons treated with OGD/reoxygenation and in vivo in rat hippocampus during cerebral ischemia-reperfusion. In all experimental schedules the enzyme is S-nitrosylated in resting or physiological state and undergoes significant denitrosylation under oxygen deprivation, which is coupled with its increased activity [75]. The subsequent increase in NO^•^ production mediates the S-nitrosylation of proteins that initiate apoptotic signals in neurons, such as GluR6, c-Jun N-terminal kinase 3, phosphatases or tensin homolog [76].

In contrast to nNOS, the process of S-nitrosylation inhibits the activity of eNOS by inducing dimer disruption, dephosphorylation and changes in the subcellular targeting status as shown in vitro [77,78].

Two of the most important proteins that undergo aberrant S-nitrosylation in response to ischemic injury are GAPDH [79,80] and matrix metalloproteinase 9 (MMP9) [81]. GAPDH has been implicated in neurotoxicity and neurodegeneration and regulates transcriptional activation, apoptosis initiation, ER to Golgi vesicle shuttling and fast axonal or axoplasmic transport [82]. Following an ischemic episode, GAPDH accumulates rapidly both in the ischemic core and in penumbral apoptotic neurons as shown in vivo using MCAO model in rats [83]. Aberrant GAPDH s-nitrosylation, translation to nucleus, concomitant neuronal death occur during the early stages of reperfusion as shown, e.g., in the rat four-vessel occlusion ischemic model [80].

MMP9 belongs to the zinc metalloproteinase family, which is involved in the degradation of the extracellular matrix. The enzyme is acutely activated during ischemia and is selectively S-nitrosylated by NO^•^ during cerebral ischemia in vivo [81]. In in vitro studies s-nitrosylation of the cysteine switch at the active site of the enzyme together with ROS-mediated oxidation of MMP9 to sulfinic or sulfonic acid derivatives triggered the apoptotic form of cell death [81], suggesting a potential extracellular proteolysis pathway to neuronal cell death in which s-nitrosylation activates MM9.

### 2.2. Hypoxia-Inducible Factor 1α (HIF-1α)

HIF-1α is the primary mammalian transcription factor specifically regulated by hypoxia and plays an essential role in cellular and systemic O_2_ homeostasis by regulating the expression of genes important in tissue survival, that regulate glycolysis, erythropoiesis, angiogenesis or catecholamine metabolism [84].

HIF is primarily regulated by changes in protein stability and transcriptional activity in oxygen-dependent manner. Under physiological conditions HIF-1α is rapidly degraded and α subunit is hydroxylated by asparagine and proline residues by the family of prolyl-4-hydroxylase domain (PHD) proteins and factor inhibiting HIF (FIH), whose activity is dependent on molecular oxygen, ferrous iron, 2-oxoglutarate [85,86,87]. In response to low tissue oxygenation during ischemic stroke, the activity of PHD and FIH declines resulting in stabilization of HIF-1α, its accumulation within the cell and translocation into the nucleus. It results in the transcriptional activation of several dozen hypoxia-responsive genes through binding the hypoxia-responsive element (HRE) in their promoter region [88,89,90,91].

The activity of HIF-1α is regulated by NO via the mechanism of S-nitrosylation. NO metabolites such as S-nitrosoglutathione (GSNO) and peroxynitrite stabilizes or destabilizes HIF-1α, respectively [92,93,94].

The impact of HIF-1α on neuronal survival upon stroke is controversial, as it mediates both anti- and pro-survival genes [85]. Up regulation of vascular endothelial growth factor (VEGF) or erythropoietin (EPO) promotes adaptation to hypoxic/ischemic stress [95] and results in reduced infarct size after cerebral ischemia in MCAO model in rodents [58,85]. The HIF-1α dependent activation of eNOS transcription and subsequent NO release in the endothelium also contribute to reduction of ischemia infarct volume also shown in MCAO model [96]. Exogenously administered NO metabolite S-nitrosoglutathione (GSNO) was found not only to stabilize HIF-1α and to induce HIF-1α-dependent genes but also to stimulate the regeneration process and to aid in functional recovery in traumatic brain injury animal model [93].

On the other hand HIF-1α regulates iNOS transcription preserving iNOS translation during ischemia, thus contributing to progressive ischemia-induced inflammation [97,98].

Tissue-specific knockouts have also given conflicting results on the role of HIF-1α during ischemia. The studies of Helton et al. showed that the brains from neuron-specific HIF-1α deficient mice were protected from hypoxia-induced cell death, when subjected to normobaric chamber or bilateral carotid artery occlusion (BCAO) model, suggesting that decreasing HIF-1α level can be neuroprotective [84]. Additionally, in HIF-1α KO mice genes involved in apoptotic pathway were uniquely downregulated when compared with WT animals. Endothelial-specific HIF-1α knock-out reduced BBB permeability and brain infarction in diabetic mice subjected to MCAO procedure [99]. In in vitro studies, in primary cortical neuron cultures, silencing HIF-1α attenuated the accumulation of iNOS [100].

On the other hand in the studies of Baranova et al. neuron-specific inactivation of HIF-1α increased brain injury in mice MCAO model [101]. Additionally, neuron-specific PHD inactivation which resulted in up-regulation of HIF-1α lead to smaller infarct size and reduced edema formation in transient MCAO model in mice [102].

In the models of permanent MCAO or acute phase of ischemic stroke in mice the expression of HIF-1α was enhanced in the place of injury, causing the massive production of iNOS [103], indicating that activation of HIF-1α might be involved in the mechanisms through which iNOS promotes cell death or survival constituting a critical factor in widespread inflammation and subsequent pathological events. Another studies showed that intermittent hypoxic conditions after brain ischemia displayed a neuroprotective effect, and despite relatively high expression of HIF-1α, lower expression of iNOS in the border between infarcts and normal tissue was observed, suggesting that overactivation of HIF-1α may suppress the activation of microglia in ischemic mice [104]. Enhanced HIF-1α activation was responsible for triggering the transcription of HIF-regulated genes (VEGF, erythropoietin, eNOS), reduced infarct size and caspase-3 activation in MCAO or common carotid arteries occlusion models in mice [96]. Increase HIF-1α was accompanied by increased iNOS expression as shown in MCAO-reperfusion injury model in rats [105].

In in vitro studies the upregulation of HIF-1α signaling was shown to improve cGMP production following ischemia through the maintenance of cGMP protein kinase activity [106], thus preventing the NO-mediated production of ROS/RNS instead of cGMP.

Overall, the role of HIF-1α in ischemia remains inconclusive, but despite controversial results the role of HIF-1α was shown to be of special importance in ischemia preconditioning and may either promote or prevent neuronal survival [107]. Partially the pro-survival and pro-inflammatory roles in the ischemic brain might depend on the injury model, time point or cell type assessed [101,108].

The contradictory results indicate that phenotype and transcriptional response to hypoxia in vivo is much more complex that would have been supposed. The brain has multiple ways of inducing HIF-1α-dependent genes involved in the response to hypoxia (that promote erythropoiesis, angiogenesis or vasodilation), and one of the alternatives is HIF-2α [109,110,111,112]. HIF-1α and HIF-2α combined KO mice exhibited reduced expression of the anti-survival genes in MCAO model in mice [85]. Even though the mice initially performed better, became more impaired 72 h after reperfusion, accompanied by increased apoptosis and reduced angiogenesis. HIF-1α and HIF-2α can partially compensate for each other, although specific target genes are differentially regulated after ischemia. Combined loss of neuronal HIF-1α and HIF-2α impairs functional recovery after cerebral ischemia, which may be beneficial predominantly in the early phase after stroke, indicating a timely regulated activation-inhibition of hypoxia-regulated cytoprotective and damaging factors which may be important for the functional outcome after stroke [85].

Another example of mutuality that may promote proapoptotic genes in response to hypoxia is p53/HIF-1α interaction [113,114,115]. Specific disruption of this interplay (attributable to the lack of HIF-1α) leads to downregulation and loss of expression of genes that promote cell death [84,98].

## 3. Treatment Strategies

As discussed above, a variety of factors contribute to the severity of stroke and its long-term consequences. Treatment strategies based on NO^•^ signaling may lower the risk of severe and irreversible complications after stroke, but they may also have detrimental effects and must therefore be provided with caution.

The advantage of NO^•^-based therapies is the possibility of modulating endogenous mechanisms activated after cerebral ischemia with exogenously applied compounds. The objective is to promote neuroprotective outcomes and integrate cellular signaling pathways at different stages of brain damage. The intervention time, pharmacokinetics, pharmacodynamics and activities of the compounds are critical to successfully counteract the consequences of stroke.

The present knowledge indicates that the therapeutic window to reduce the pathological consequences of stroke, essentially neuronal damage, is estimated as 0–6 h for primary interventions [116,117] and may extend up to 24 h poststroke.

As discussed above, NO-mediated actions clearly indicate that the activation of eNOS contributes to proper vasodilation, exerts antioxidant, anti-inflammatory and anti-atherogenic effects, and regulates glucose uptake and insulin sensitivity [118,119], thus exerting protective effects in stroke. In contrast, the inhibition of nNOS and iNOS, the main generators of free radicals, alternatively elimination of free radicals, may counteract neurodegeneration.

### 3.1. Free Radical Scavengers

Reperfusion of ischemic areas can exacerbate ischemic brain damage through the generation of ROS/RNS (e.g., O_2_^•^^−^, hydroxyl radicals and ONOO^−^) by excessive production of both NO^•^ and O_2_^•^^−^ by nNOS and iNOS. It has been reported that excess of NO^•^ immediately reacts with O_2_^•^^−^ to form ONOO^−^ [120,121], which is responsible for the nitration of both free and protein bound tyrosine residues which are known to disrupt cell signaling cascades leading to tissue injury [120,121]. Therefore, enhanced degradation of ROS/RNS with pharmacological agents has been found to limit the extent of brain damage following stroke-induced excess of NO^•^ generation. Elimination of free radicals attenuates cytokine formation that drives up regulation of inflammatory proteins including iNOS.

On the other hand ROS provides a redox signal for hypoxic HIF-1α activation [122]. Assuming, that under specific pathological conditions, increased activity of HIF-1α is responsible for ischemia-induced detrimental effects, application of anti-oxidants eliminates ROS and consequently reduces HIF-1α levels by its destabilization and loss of transcriptional activity [123].

Under normal conditions, endogenous protective enzyme systems, such as superoxide dismutase (SOD) and reduced glutathione (GSH), limit the overproduction of free radicals. However, their capacities may be overwhelmed by pathological changes when free radical generation is uncontrolled. Removal of pathologically produced free radicals may be regarded as a viable approach to neuroprotection and may be achieved by scavenging or trapping free radicals. Among the compounds that possess free radical scavenging properties are nitrones, thiols, iron chelators, phenols and catechols. Tirilazad, ebselen and edaravone are examples of compounds with scavenging activity, and compounds with free radical trapping properties include NXY-059 and NSP-116.

Edaravone (Radicut) is a free radical scavenger marketed in Japan to treat acute ischemic stroke [124]. Edaravone scavenged •HO, NO^•^ and ONOO^−^ in a concentration-dependent manner [125,126,127]. The efficacy of the drug ranges from large clinical improvements to modest effects measured with standard stroke scales when administered up to 72 h following ischemic stroke [128,129].

Tirilazad mesylate, a lazaroid, has been investigated as a neuroprotective agent in patients after acute ischemic stroke. The compound was ineffective in treating acute ischemic stroke [130,131]. Another seleno-organic compound, ebselen, was shown to reduce delayed ischemic neurological deficits after SAH [132] and to improve outcomes after stroke [133,134].

### 3.2. Enhancement of NO Production

L-arginine, a substrate for NO^•^ biosynthesis, is an obvious candidate of choice to improve NO bioavailability. The administration of L-arginine increased regional blood flow and prevented tissue damage in the rat MCAO model [135,136,137]. However, contradictory results with L-arginine administration were obtained that showed a beneficial effect [135,136,137], no effect [138] and the potentiation of pathological changes [139,140]. These effects may result from the fact that L-arginine enhances NO^•^ synthesis via all three isoforms [141]; therefore, synthesis of potentially detrimental NO^•^ from iNOS or nNOS may counteract the neuroprotective effects of eNOS activation. The ability of L-arginine to stimulate the release of hormones such as insulin [142], glucagon [143], growth hormone [144] or catecholamines [145] and its ability to transform into toxic byproducts such as polyamines or agmatine [146,147,148,149] are also limitations of its use.

NO^•^ donors, such as organic nitrates, sodium nitroprusside (SNP), sydnonimines, S-nitrosothiols, NONOates and hybrid donors potentiate NO^•^ production and thus may potentially be regarded as treatment strategies.

Administration of DETA/NONOate [150], SNP [151,152], 3-morpholinosydnonimine [153,154], ZJM-289 [155] or LA-419 [156,157] significantly increased cell proliferation and/or reversed ischemia-induced tissue damage in the selected structures of the rat brain. Stimulation of eNOS activity with concomitant suppression of nNOS and iNOS function was observed [155,156,157]. Beyond this point, neurovascular toxicity instead of vasodilation or infarct size reduction was observed [152].

These results from preclinical studies were confirmed in clinical observations in which SNP reduced the mean arterial blood pressure and led to improvements in CBF in patients after acute stroke [158,159].

The mechanism of the neuroprotective action of NO^•^ donors includes their abilities to reduce oxidative stress both in the brain and blood, inhibit the expression and activities of MMPs, scavenge NO^•^ or quench ROS, reduce inflammation, exert antiplatelet effects and attenuate I/R-mediated increases in ICAM-1 and E-selectin mRNA expression [160,161].

Suppression of the hydrolysis of L-arginine into ornithine and urea by arginases increased the production of NO^•^ and prevented the development of endothelial dysfunction [162], similar to the effects of eliminating asymmetric dimethylarginine (ADMA)—endogenous NOS inhibitor, through the stimulation of ADMA-hydrolyzing enzyme (DDAH II) [163].

The available data concerning the efficacy of the enhancement of NO^•^ production-based therapies are collected in Table 1.

### 3.3. NOS Inhibitors

Selective inhibition of nNOS and iNOS activities is thought to counteract the aftermath of stroke.

The neuroprotective abilities of N(ω)-nitro-L-arginine methyl ester (L-NAME) and 7-nitroindazole (7-NI), widely used NOS/nNOS inhibitors, are time-dependent and are observed in the early stages of ischemic insult [164]. The choice of dose is important, as the compounds prevent at low doses but potentiate ischemia-induced neurodegeneration at high doses [165]. Long-term inhibition of NOS might be too risky because of the off-target effects on eNOS, particularly in patients with cardiovascular risk or metabolic diseases.

Direct or indirect inhibition of iNOS through selective inhibitors, such as aminoguanidine (AG) or the BH_4_ rate-limiting enzyme GTP cyclohydrolase I, attenuated cerebral infarction, ischemia-induced pathologies and prevented the progression of cerebral aneurysms [166,167,168].

The available data concerning the efficacy of NOS inhibitor-based therapies are collected in Table 1. For review please see also: [164].

**Table 1:** Enhancement of NO^•^ production and NOS inhibitor-based therapies. Pretreatment indicates that the compound was administered before experimental ischemia; posttreatment indicates that the compound was administered after the episode. MCAO—middle cerebral artery occlusion, ODG—oxygen and glucose deprivation.

	Compound	Effect	
**NO Donors**			
Pretreatment	LA-419	in vivo: − reduced iNOS, nNOS, nitrotyrosine expression and increased apparent diffusion coefficient in endothelin-1-induced focal cerebral ischemia or global cerebral ischemia model in rats induced by oxygen and glucose deprivation	[156,157]
	GSNO	in vivo: − reduced caspase-3, -8, -9, tBID cleavage in global ischemia models in rats − increased Bid, pro-caspase-3 and pro-caspase-9 expression in global ischemia model in rats − reduced Fas, CaMKII, MKK4 S-nitrosylation, increased nNOS S-nitrosylation and phosphorylation, increased CaMKII phosphorylation in global ischemia models in rats − increased cell density in CA1 in global ischemia models in rats − reduced infarct size and edema in MCAO model in mice	[169,170,171,172]
	ZJM-289	in vitro: − increased cell viability in primary cortical neuron in OGD model − reduced mitochondrial dysfunction in primary cortical neuron in OGD model − decreased Ca^2+^ release and ROS production in primary cortical neuron in OGD model in vivo: − reduced infarct size and edema and improved neurologic deficit in MCAO model in rats	[155,173]
	SIN-1	in vivo: − no influence or reduced infarct size in MCAO model in rats	[174,175]
	DETA NONOate	in vivo: − no influence on infarct size in MCAO model in rats	[174]
	NBP	in vitro: − increased cell viability in primary cortical neuron in OGD model − reduced mitochondrial dysfunction in primary cortical neuron in OGD model − decreased Ca^2+^ release and ROS production in primary cortical neuron in OGD model in vivo: − reduced infarct size and improved neurologic deficit in MCAO model in rats	[173,176]
	Spermine NONOate	in vivo: − reduced infarct size in MCAO model in rats − increased cortical perfusion in MCAO model in rats	[152]
	sodium nitroprusside	in vivo: − reduced infarct size in MCAO model in rats	[152]
Posttreatment	GSNO	in vivo: − reduced infarct size	[171]
	DETA NONOate	in vivo: − no influence on infarct size in MCAO model in rats − improved neurologic deficit and increased cGMP level in MCAO model in rats − increased cell proliferation in subventricular zone, olfactory bulb and dentate gyrus in MCAO model in rats	[150]
	SIN-1	in vivo: − reduced infarct size in MCAO model in rats	[153,154]
	sodium nitroprusside	in vivo: − reduced infarct size in MCAO model in rats	[153]
**NOS or nNOS inhibitors**			
Pretreatment	7-NI	in vivo: − increased nNOS S-nitrosylation and phosphorylation, decreased CaMKII and MKK4 S-nitrosylation and increased CaMKII phosphorylation in global ischemia model or MCAO in rats − decreased caspase-3 cleavage in MCAO model in rats − increased cell density in CA1 in global ischemia model or MCAO model in rats − reduced maximal NO concentration in bilateral common carotid artery occlusion in rats	[170,172,177]
	L-NAME	in vivo: − reduced infarct size and improved neurological deficit in MCAO model in rats − reduced glutamate, aspartate, glutamine synthetase and nitrate/nitrite level in MCAO model in rats − increased ATP and NAD level in MCAO model in rats − reduced TNF-α expression and increased IL-10 expression in MCAO model in rats	[174,178]
Posttreatment	7-NI	in vivo: − reduced neuronal damage in global cerebra ischemia in rats	[179]
	L-NAME	in vivo: − reduced infarct volume in MCAO model in rats and mice − improved neurological deficit in MCAO model in rats and mice − reduced level of tissue nitric oxide end products in MCAO model in mice − reduced nitrate/nitrite level and increased NAD level in MCAO model in rats	[178,180]
**iNOS inhibitors**			
Pretreatment	aminoguanidine	in vivo: − reduced infarct volume, edema, neurological deficits, necrotic cell death in penumbra and core and reduced apoptosis in penumbra in MCAO model in rats	[181]
Posttreatment	aminoguanidine	in vivo: − reduced infarct volume in permanent MCAO model in mice − reduced ischemia-induced neurogenesis in dentate gyrus in MCAO model in rats	[167,182,183]
	1400 W	in vivo: − reduced infarct size neurological deficit in MCAO model in rats − inhibited delayed increase in glutamate level in MCAO model in rats	[184]
	S-methylisothiorea	in vivo: − reduced neurological deficit, mortality, infarct volume ratio in MCAO model in rats − attenuated morphological changes in cortical neurons in MCAO model in rats	[185]

### 3.4. HIF-1α

HIF-1α is an essential component in changing the transcriptional repertoire of tissues during oxygen deprivation and plays a pivotal role in the regulation of iNOS activity. Thus, HIF-1α and the genes regulated by it have been the center of intense research. A growing number of pre-clinical studies in rodents suggests that the activation of HIF-1α signaling pathway prior or shortly after ischemic stroke reduces tissue damage and increases functional recovery from ischemic stroke [87,96,186,187,188,189]. One example of an agent that stabilizes the transcriptional activator HIF-1α and activates target genes involved in compensation for ischemia are inhibitors of HIF-1α prolyl hydrolases (PHD1, PHD2 and PHD3) [190]. The beneficial effects of PHD inhibition after ischemia require the activity of HIF-1α as shown in in vitro in oxygen glucose deprivation model of ischemia and in vivo in MCAO model in mice [96]. Dimethyloxalylglycine (DMOG) enhanced the activation of HIF-1α and enhanced transcription of the HIF-regulated genes. In vivo the infarct size, activation of pro-apoptotic proteins and behavioral deficits after stroke were reduced. The effect of DMOG was decreased after inhibition of HIF-1α with digoxin [96]. Similar effects were observed with small molecule hypoxia mimics, such as deferoxamine, cobalt chloride or GSNO. Additionally, these agents increased the expression of HIF-1α target genes [92,93,191]. Cocaine, andrographolide or vitamin E activated the HIF-VEGF pathway, thus increasing microvascular density, restoring local blood flow and protecting the brain from ischemic insults [192,193,194]. However, available data indicate that sustained and prolonged activation of the HIF-1α pathway may lead to a transition from neuroprotection to neurodegeneration, reflecting the dual features of the HIF system [195,196,197], which should be taken into consideration when considering therapy to modify HIF-1α. Some contradictory results indicated that inhibition of HIF-1α improved brain function in ischemia-reperfusion brain injury-related disorders [105,198]. Inhibition of free radical formation, followed by inhibition of HIF-1α activation, apoptosis formation, neutrophil activation and iNOS expression resulted in reduction in the infarct volume in ischemia-reperfusion brain injury (MCAO model) in rats [105]. In rat model of transient MCAO the early inhibition of HIF-1α by application of inhibitors or small interfering RNA reduced infarct size and BBB hyperpermeability, decreased mortality and improved neurological deficits through inhibition of HIF-1α activity [198,199]. The studies of Hsiao et al. showed that the administration of PMC [105].

The therapeutic strategies might lead to pleiotropic activation of HIF signaling through all cell types in CNS, making it difficult to draw conclusions about the significance of the HIF signaling pathway in the treatment of ischemia-reperfusion injury. However, the stimulation of the non-ischemic penumbra regions to initiate HIF-1α (e.g., by inhibition of PHD) and subsequent downstream induction of HIF-1α mediated antiapoptotic, vascular and glycolytic metabolic changes before the area is enveloped by the spreading ischemia may be one of the proposed therapeutic HIF-dependent effect. Additionally, inhibition of endothelial HIF-1α warrants further investigation as a therapeutic target for the treatment of stroke patients with diabetes [99].

The available data concerning the efficacy of HIF-1α-based therapies are collected in Table 2.

### 3.5. Combination Therapies

At present, it seems that combination therapies are more effective for treating stroke than monotherapies. Scavenging reactive oxygen species and concomitantly inhibiting NO^•^ synthesis by administering statins with resveratrol, an approved antioxidant or with nifedipine, a calcium channel blocker, is just one example of combination therapy for stroke [226]. Administration of resveratrol upregulated antioxidant enzyme activities, decreased O_2_^•^^−^ production by downregulating NADPH oxidase activity and attenuated oxidative stress-mediated eNOS uncoupling. Some hope may be placed in combination therapy, which could allow minimization of the dose of NMDA antagonists by administering a compound enhancing its effect, such as caffeine [227] or cordycepin (an adenosine analog) [228]. The use of different agents at the same time enables avoidance of detrimental consequences induced by undesired side effects and reinforcement of beneficial impacts by mutual action. The examples of effective combined treatment therapies in clinical trials are collected in Table 3.

## 4. Conclusions

Stroke is one of the most common indications with unmet medical needs in medicine, and it is a major challenge to develop effective treatments, as stroke treatments need to reduce cell death and infarct size, stabilize the blood-brain barrier, reduce reoxygenation-induced leakage and preserve neuromotor function in a supra-additive manner. The NO^•^-dependent pathway plays a crucial role in all processes involved in subsequent events after ischemia [49,154,235]. However, NO^•^-based treatments can be burdened with a variety of adverse effects. The risk of exacerbating pathological changes with NO^•^-regulating agents is also high. The objective is to restore the imbalance L-arginine/ADMA-NOS-NO^•^ [236] and protect against hypoxic/ischemic-derived damage.

The use of combined treatment therapies seems to be the best alternative. The number of plausible combinations is almost unlimited. The only limitation is the satisfactory effectiveness and the lack of the induction of adverse effects. The challenge is to find satisfactory solution. NO^•^-related pathways and the agents that inhibit excess of NO^•^ production especially of that which comes from nNOS or iNOS, free radicals’ scavengers or compounds targeting HIF-1α give a number of possibilities that still have not been fully investigated so far. The vast range of studies discussed in the present review and by others indicates, that the area is still open for investigation.

## Figures and Tables

**Table 2:** HIF-1α-based therapies. Pretreatment indicates that the compound was administered before experimental ischemia; posttreatment indicates that the compound was administered after the episode. MCAO—middle cerebral artery occlusion, ODG—oxygen and glucose deprivation.

	Compound	Effect	
**HIF-1α Stabilizing Agents**			
Pretreatment	Deferoxamine (DFO)-iron chelator	in vivo: − reduced infarct volume in animal models of focal and global ischemia in vitro: − tolerance against oxygen-glucose deprivation in purified cortical neurons	[101,187,200,201,202,203]
	Cobalt chloride (CoCl_2_)	in vivo: − reduced brain injury, and infarct size in newborn rat model of ischemia	[200,203,204]
	Prolyl hydroxylase (PHD) inhibitors − PHD1 − PHD2 − PHD3	in vivo: − reduced brain infarct size, reduced post-stroke neurological deficit, reduced cognitive dysfunction and decreased formation of vasogenic edema in transient or permanent MCAO model in rats or mice − attenuated neuronal death and reactive astrogliosis by 20–50% in transient bilateral common carotid artery occlusion ischemia model in gerbils in vitro: − reduced cell death (OGD, PC12 cell line and primary rat cortical neurons) − reduced number of early apoptotic cells (OGD, PC12 cell line)	[87,186,188,189,205,206,207]
Posttreatment	Prolyl hydroxylase inhibitors − PHD1 − PHD2 − PHD3	in vivo: − reduced brain infarct size in MCAO model in rats − attenuated neuronal death and reactive astrogliosis by 20–50% in transient bilateral common carotid artery occlusion ischemia model in gerbils in vitro: − stabilized HIF-1α and up-regulated HIF-1 dependent target genes in primary murine astrocytes and murine cerebrovascular endothelial cell line (bEnd.3) in normal conditions − protective effects in oxygen-glucose deprivation in mouse hippocampal neuronal HT-22 cell line	[188,205]
**HIF-1α dependent proteins**			
Pretreatment	Erythropoietin (EPO)	in vivo: − protection against ischemia-induced cell death in bilateral common carotid artery occlusion in gerbils − reduction of infarct volume in permanent MCAO model in mice or transient MCAO model in rats	[208,209,210,211]
Posttreatment	Erythropoietin (EPO), or its analogs (MEPO, S104I-EPO)	in vivo: − neuroprotective effect in transient MCAO model in rats up to 6 h posttreatment − enhancement of neuronal survival in stroke-prone hypertensive rats after permanent MCAO − enhancement of angiogenic responses in rat model of neonatal ischemia (permanent right CCAO) − reduced perihematomal inflammation and apoptosis, induced functional recovery and upregulation of eNOS, STAT3, ERK in intracerebral hemorrhage model in rats in vitro: − reduced NMDA-induced excitotoxicity in primary cortical neurons − reduced cell death after hypoxia in rat primary hippocampal neurons	[211,212,213,214,215]
	Vascular endothelial growth factor (VEGF)	in vivo: − reduced infarct size, enhanced neurogenesis, angiogenesis, cerebral microvascular perfusion and neurological dysfunction in MCAO and focal cerebral embolic ischemia in rats	[216,217]
**Direct and indirect HIF-1α inhibitors**			
Pretreatment	Acriflavine	in vivo: − increased neurological deficit in endothelin-1-induced focal cerebral ischemia in mice − no influence in infarct volume, number of neurons, number of IL-10-positive cells, iNOS expression and pro-inflammatory cytokines endothelin-1-induced focal cerebral ischemia in mice − decreased expression of HIF-1α and increased expression of NF-κB endothelin-1-induced focal cerebral ischemia in mice − increased number of GFAP-positive cells and GFAP expression endothelin-1-induced focal cerebral ischemia in mice	[218]
	2,2,5,7,8-Pentamethyl-6-hydroxychromane (PMC)	in vivo: − reduced infarct volume in MCAO model in rats − reduced caspase-3 activation, reduced HIF-1α, iNOS and nitrotyrosine expression in MCAO model in rats	[105]
	YC-1	in vivo: − reduced HIF-1α, VEGF, EPO and GLUT-3 expression in MCAO model in rats − increased mortality, infarct size and edema in MCAO model in rats − reduced BBB permeability in MCAO model in rats	[219]
	Chrysin	in vivo: − reduced oxidative stress biomarkers in hippocampus in bilateral carotid artery occlusion in rats − reduced TNF-α, IL-6, BAX and Hsp90 level in hippocampus in bilateral carotid artery occlusion in rats − increased IL-10 and Bcl-2 level in hippocampus in bilateral carotid artery occlusion in rats − increased aspartate and glutamate level in hippocampus in bilateral carotid artery occlusion in rats − reduced infarct volume and neurological deficit in MCAO model in mice − reduced SOD activity and malondialdehyde level in MCAO model in mice − reduced number iNOS-, COX2- and NF-κB-positive cells and their protein expression in MCAO model in mice − reduced number of GFAP- and Iba-1-positive cells in MCAO model in mice	[220,221]
Posttreatment	2ME2	in vivo: − reduced infarct volume and edema in neonatal model of ischemia or MCAO model in rats − reduced HIF-1α and VEGF expression in neonatal model of ischemia − reduced mortality in MCAO model in rats − increased neurological score in MCAO model in rats − reduced HIF-1α expression in MCAO model in rats − reduced density of HIF-1α-, VEGF-, BNIP3- and caspase-3-positive cells in MCAO model in rats	[199,222]
	D609	in vivo: − reduced infarct size and mortality in MCAO model in rats − increased neurological score in MCAO model in rats − reduced density of HIF-1α expression in MCAO model in rats − reduced HIF-1α-, VEGF-, BNIP3- and caspase-3 positive cells in MCAO model in rats	[199]
	Chetomin	in vivo: − increased infarct size and neurological deficit in MCAO model in rats	[223]
**Clinical trials**			
	rhEPO (double-blind placebo controlled proof-of-concept trial; i.v.)	− better clinical recovery − normalization of circulating marker of injury (S100β)	[224]
	EPO (prospective, randomized, placebo-controlled trial; s.c.)	− improved long-term clinical outcome	[225]

**Table 3:** Combined treatment therapies with the use of NO-related compounds in clinical trials. SOC—standard of care.

Mode of Action	Combined Treatment	Effect	
Free radical scavenger	Edaravone + Hyperbaric oxygen + Heparin (pilot trial)	- reduction of neurological symptoms (NIHSS)	[229]
	Edaravone + t-PA (pilot trial)	- early recanalization - higher percent of remarkable recovery (≥8-point reduction on NIHSS)	[230]
	Edaravone + t-PA (multicenter, prospective, randomized and open-label trial)	- no effect on the rate of early recanalization, symptomatic intracerebral hemorrhage or favorable outcome after tPA therapy	[231]
NMDA antagonist, VGSC and VGCC blocker, inhibitor of NO synthesis	Lubeluzole + t-PA (feasibility, safety and efficacy trial—uncompleted)	- study was terminated after Lubeluzole’s phase III trial showed no overall improvement	[232]
Statin	Simvastatin + t-PA (phase IV, prospective, randomized, double-blind, placebo-controlled trial)	- higher proportion of patients undergo major neurological recovery (post hoc analysis)	[233]
Typical antipsychotic + First-generation antihistamine	Chlorpromazine + Promethazine + SOC (pilot trial)	- no effect on neurological symptoms (NIHSS, mRS)	[234]
